# Platelet-rich plasma versus autologous blood versus steroid injection in lateral epicondylitis: systematic review and network meta-analysis

**DOI:** 10.1007/s10195-015-0376-5

**Published:** 2015-09-11

**Authors:** Alisara Arirachakaran, Amnat Sukthuayat, Thaworn Sisayanarane, Sorawut Laoratanavoraphong, Wichan Kanchanatawan, Jatupon Kongtharvonskul

**Affiliations:** Orthopedics Department, Police General Hospital, Bangkok, Thailand; Orthopedics Department, Lerdsin General Hospital, Bangkok, Thailand; Section for Clinical Epidemiology and Biostatistics, Faculty of Medicine, Ramathibodi Hospital, Bangkok, Thailand

**Keywords:** Lateral epicondylitis, PRP, Autologous blood, Corticosteroid, Systematic review, Network meta-analysis

## Abstract

**Background:**

Clinical outcomes between the use of platelet-rich plasma (PRP), autologous blood (AB) and corticosteroid (CS) injection in lateral epicondylitis are still controversial.

**Materials and methods:**

A systematic review and network meta-analysis of randomized controlled trials was conducted with the aim of comparing relevant clinical outcomes between the use of PRP, AB and CS injection. Medline and Scopus databases were searched from inception to January 2015. A network meta-analysis was performed by applying weight regression for continuous outcomes and a mixed-effect Poisson regression for dichotomous outcomes.

**Results:**

Ten of 374 identified studies were eligible. When compared to CS, AB injection showed significantly improved effects with unstandardized mean differences (UMD) in pain visual analog scale (VAS), Disabilities of Arm Shoulder and Hand (DASH), Patient-Related Tennis Elbow Evaluation (PRTEE) score and pressure pain threshold (PPT) of −2.5 (95 % confidence interval, −3.5, −1.5), −25.5 (−33.8, −17.2), −5.3 (−9.1, −1.6) and 9.9 (5.6, 14.2), respectively. PRP injections also showed significantly improved VAS and DASH scores when compared with CS. PRP showed significantly better VAS with UMD when compared to AB injection. AB injection has a higher risk of adverse effects, with a relative risk of 1.78 (1.00, 3.17), when compared to CS. The network meta-analysis suggested no statistically significant difference in multiple active treatment comparisons of VAS, DASH and PRTEE when comparing PRP and AB injections. However, AB injection had improved DASH score and PPT when compared with PRP injection. In terms of adverse effects, AB injection had a higher risk than PRP injection.

**Conclusions:**

This network meta-analysis provided additional information that PRP injection can improve pain and lower the risk of complications, whereas AB injection can improve pain, disabilities scores and pressure pain threshold but has a higher risk of complications.

**Level of evidence:**

Level I evidence

## Introduction

Lateral epicondylitis is the most commonly diagnosed condition of the elbow [21], with a prevalence of 1–3 % in the general population [34]. It affects men and women equally, mainly in the age range of 35−55 years [2, 28]. In most cases of lateral epicondylitis, no obvious underlying etiology can be identified [25]. However, any activity that involves overuse of the wrist extensor or supinator muscles may be incriminating. The most commonly affected muscle is the extensor carpi radialis brevis (ECRB), as originally described by Cyriax [2]. The pathology of lateral epicondylitis was previously considered to be from tendinitis, arising as inflammation of the tendon [18]. Histopathologically, it has been shown to have a paucity of inflammatory cells such as macrophages and neutrophils [7, 11]. The condition is therefore considered to be a form of tendinosis, which is defined as a degenerative process [2]. The treatment of lateral epicondylitis includes rest, nonsteroidal anti-inflammatory medication, bracing, physical therapy, extracorporeal shock wave therapy and botulinum toxin injection. Injection of corticosteroids (once the gold standard but now considered controversial), whole blood and platelet-rich plasma (PRP), and various types of surgical procedures have also been recommended [4, 8, 17, 27, 29, 35]. Injection with corticosteroids has been used since the 1950s and has been the treatment of choice for many years. However, several studies have shown no long-term beneficial effect; several alternative biologic injection therapies have therefore become available. Complex growth factor preparations, derived from the patients’ own (autologous) blood, are used to drive the body’s own tissue-healing mechanisms in the hope of stimulating rapid healing mechanisms [5]. Two different preparations that are most described in the literature are autologous whole blood (AB) and platelet-rich plasma (PRP) injection [5, 10, 12, 14, 19, 21, 23, 24, 26, 33]. There have been several randomized controlled trials (RCTs) that have compared AB with PRP injection [5, 23, 24, 33], AB with steroid injection[12, 26] and PRP with steroid injection [10, 14, 19, 21]. However, results as to whether PRP, AB or corticosteroids is more beneficial are still unclear. Previous systematic reviews by Krogh et al. [13] including 17 studies have shown eight different injection therapies reported by network meta-analysis. The results showed that AB, PRP and corticosteroids were more efficacious than placebo [estimated by standardized mean difference (SMD)]; however, there were no reports comparing the efficacy of PRP versus AB, PRP versus corticosteroids and AB versus corticosteroids. Ahmad et al. [1] showed that PRP was more efficacious than blood injection in terms of non-response rate and conversion to surgery rates as well as pain visual analog score (VAS), and that PRP was more efficacious than corticosteroid injections in terms of pain and Disabilities of the Arm, Shoulder and Hand (DASH) score in only one of three studies, but two other studies showed no clinically significant difference. However, these meta-analyses included too few studies for pooling of the outcomes, utilized standardized mean difference, and lacked proper methodological quality required for performing a network meta-analysis. Neither heterogeneity nor sources of heterogeneity (age, sex, disease duration, preparation of the intervention and time to assess the outcome) were assessed. Moreover, other RCTs [23, 24, 26] have been published since this study was done. Therefore, a systematic review was conducted with a network meta-analysis of RCTs at multiple follow-up times with the aim of comparing relevant clinical outcomes [visual analog score, DASH score, Patient-Related Tennis Elbow Evaluation (PRTEE) score, adverse effects and non-response rates] between AB, PRP and corticosteroids.

## Materials and methods

### Search strategy

The Medline and Scopus databases were used to identify relevant studies published in English from the date of inception to January 18, 2015. The PubMed and Scopus search engines were used to locate studies using the following search terms: ‘lateral epicondylitis’ and ‘platelet-rich plasma’ and ‘clinical trial’. Relevant studies from the reference lists of identified studies and previous systematic reviews were also explored.

### Selection of studies

**Fig. 1:**
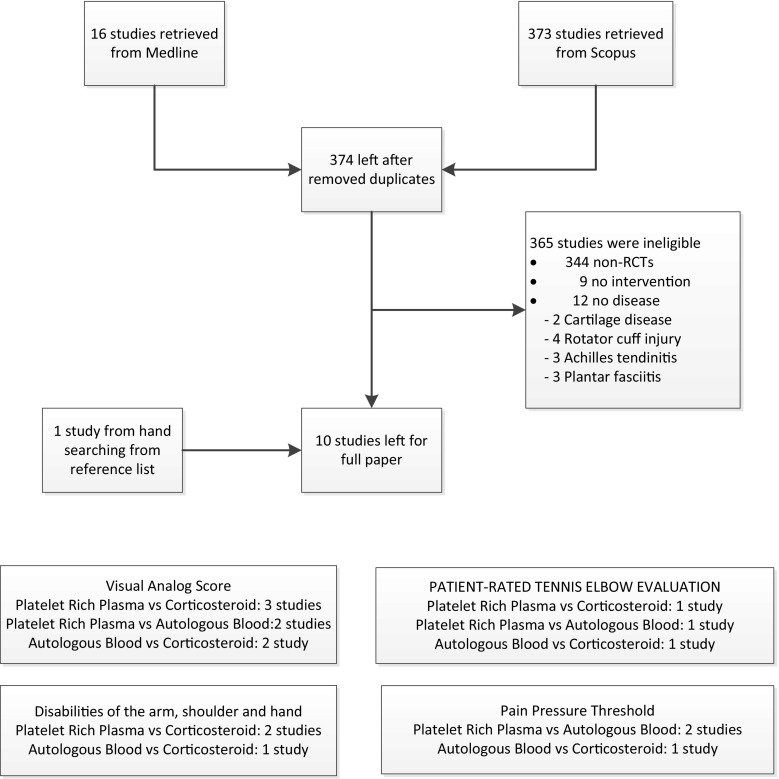
Flow-chart of study selection

Identified studies were selected by one author (J.K.) and randomly checked by A.A. Their titles and abstracts were initially screened; full papers were then retrieved if a decision could not be made from the abstracts. The reasons for ineligibility or exclusion of studies were recorded and described (Fig. 1).

### Inclusion criteria

Randomized controlled trials or quasi-experimental designs comparing clinical outcomes between treatments in lateral epicondylitis patients were eligible if they met the following criteria:

- compared clinical outcomes between PRP, AB and corticosteroid injection
- compared at least one of the following outcomes: visual analog score, DASH score, PRTEE score, pressure pain threshold (PPT), adverse effects and non-response rates
- had sufficient data to extract and pool, namely reported mean, standard deviation (SD), and numbers of subjects according to treatments for continuous outcomes; number of patients according to treatment for dichotomous outcomes

### Data extraction

Two reviewers (J.K. and A.A.) independently performed data extraction using standardized data extraction forms. General characteristics of the subjects (e.g., mean age, gender, dominant side, duration of disease, pain score, disabilities scores and PPT at baseline) were extracted. The number of subjects, mean and SD of continuous outcomes, namely pain by VAS, DASH score, PRTEE score and PPT between groups, were extracted. Cross-tabulated frequencies between treatment and adverse effects were also extracted. Any disagreements were resolved by discussion and consensus with a third party (S.L.).

### Risk of bias assessment

Two authors (J.K. and T.A.) independently assessed the risk of bias for each study. Six study quality domains were considered, namely sequence generation, allocation concealment, blinding (participant, personnel, and outcome assessors), incomplete outcome data, selective outcome reporting, and other sources of bias [15]. Disagreements between two authors were resolved by consensus and discussion with a third party (A.T.).

### Outcomes

The outcomes of interest were pain VAS, DASH score, PRTEE score, complications and non-response rates. Methods of measurements of these outcomes were used according to the original studies. Briefly, this includes the VAS pain scale of 0–10, the DASH score which consists of 30 items with total scores ranging from 0 to 100, the PRTEE which consists of pain disability and functional disability with a total score ranging from 0 to 100, and pressure pain threshold (PPT) which was assessed by an algometer with scale units in kg/cm^2^. Postoperative adverse effects (skin reaction and local injection site pain) and non-response rates were considered.

### Statistical analysis

Direct comparisons of continuous outcomes measured at the end of each study between PRP, AB and corticosteroid injection were pooled using an unstandardized mean difference (UMD). Heterogeneity of the mean difference across studies was checked using the *Q*-statistic and the degree of heterogeneity was quantified using the *I*^2^ statistic. If heterogeneity was present as determined by a statistically significant *Q*-statistic or by *I*^2^ > 25 %, the UMD was estimated using a random effects model; otherwise a fixed effects model was applied.

For dichotomous outcomes, a relative risk (RR) of adverse effect of treatment comparisons at the end of each study was estimated and pooled. Heterogeneity was assessed using the previous method. If heterogeneity was present, the Dersimonian and Laird method [3] was applied for pooling. If not, the fixed effects model by inverse variance method was applied. Meta-regression was applied to explore the source of heterogeneity (e.g., mean age, percentage of females, duration of disease, dominant hand side and follow-up time) if data was available. Publication bias was assessed using contour-enhanced funnel plots [20, 22] and Egger tests [9].

For indirect comparisons, network meta-analyses were applied to assess all possible effects of treatment if summary data was available for pooling [16, 30, 31]. A linear regression model, weighted by inverse variance, was applied to assess the treatment effects for continuous outcomes. For postoperative complications, a mixed-effect Poisson regression was applied to assess treatment effects [16]. Summary data was expanded to individual patient data using the “expand” command in STATA. Treatment was considered as a fixed effect whereas the study variable was considered as a random effect in a mixed-effect model. The pooled RR and its 95 % confidence intervals (CIs) were estimated by exponential coefficients of treatments. All analyses were performed using STATA version 13.0 [32]. *P* < 0.05 was considered statistically significant, except for the test of heterogeneity where *P* < 0.10 was used.

## Results

Sixteen and 373 studies from Medline and Scopus were identified, respectively; 15 studies were duplicates, leaving 374 studies for review of titles and abstracts. Of these, nine studies [5, 12, 14, 19, 21, 23, 24, 26, 33] plus one study [6] identified from reference lists were reviewed, leaving a total of ten studies for data extraction. Characteristics of the 10 studies [5, 6, 12, 14, 19, 21, 23, 24, 26, 33] are given in Table 1. Of seven PRP studies [5, 14, 19, 21, 23, 24, 33], the comparators included AB in four studies [5, 23, 24, 33], and steroids in three studies [14, 19, 21]. All three studies regarding AB were in comparison with steroids. Most studies [5, 6, 14, 21, 23, 26, 33] assessed outcomes at more than 2 months; only three studies [12, 19, 24] assessed outcomes at 1.5–2 months. Mean age, dominant side, duration of disease and VAS before treatment varied from 34 to 50 years, 57 to 85 %, 5 to 18 months and 5.5 to 7.6, respectively. The percentage of males ranged from 18 to 57 %. Various outcomes were compared between treatment groups (Fig. 1).

**Table 1:** Characteristics of included studies

References	Years	Journal	Intervention	Comparator	Follow-up (months)	Preparation of intervention	Age (years)	Sex (male %)	Dominant side (%)	Duration (months)	VAS (0–10) before treatment	Outcome
Kazemi et al. [12]	2010	AJPMR	AB	Steroid	2	Mixed blood with 1 ml of 2 % lidocaine	47.1	18	60	–	6.60	VAS, DASH, PPT, adverse effect
Peerboom et al. [21]	2010	AMJ	PRP	Steroid	12	The GPS III system	47.1	48	63	–	6.80	VAS, DASH, adverse effect
Thanasas et al. [33]	2011	AMJ	PRP	AB	6	The GPS III system	36.3	25	86	5.1	6.05	VAS, adverse effect
Dojode [6]	2012	BJR	AB	Steroid	6	Mixed blood with 1 ml of 0.5 % bupivacaine	42.5	42	85	8.6	7.6	VAS, adverse effect
Omar et al. [19]	2012	Egypt Rheumatologist	PRP	Steroid	1.5	Other system	37.5	37	60	1.8	8.40	VAS, DASH
Singh et al. [26]	2013	J Health Allied Sci	AB	Steroid	3	Mixed blood with 1 ml of 2 % lidocaine	34.1	47	57	18.1	–	PRTEE
Krogh et al. [14]	2013	AMJ	PRP	Steroid	3	The Recover GPS II system	45.8	50	80	–	5.55	PRTEE, adverse effect
Creaney et al. [5]	2014	BJSM	PRP	AB	6	Other system	50.1	56.5	–	–	–	PRTEE
Raeissadat et al. [23]	2014	BMC Sports Science	PRP	AB	12	The Rooyagen kit	43.5	23	67	–	6.95	VAS, PPT
Raeissadat et al. [24]	2014	Pain Research and Treatment	PRP	AB	2	The Rooyagen kit	46.3	40	65	–	7.00	VAS, PPT

*AB* autologous blood injection, *Steroid* corticosteroid, *PRP* platelet-rich plasma, *VAS* visual analog score, *DASH* Disabilities of the Arm Shoulder and Hand, *PRTEE* Patient-Rated Tennis Elbow Evaluation, *PPT* pressure pain threshold

### Risk of bias in included studies

The risk of bias assessment is described in Table 2.

**Table 2:** Risk of bias assessment

References	Adequate sequence generation	Adequate allocation concealment	Blinding	Address incomplete outcome data	Selective outcome report	Free of other bias	Description of other bias
Kazemi et al. [12]	Y	N	Y	N	Y	N	Did not mention to ITT
Peerboom et al. [21]	Y	Y	Y	Y	Y	Y	–
Thanasas et al. [33]	Y	N	Y	Y	Y	Y	–
Dojode [6]	U	N	N	Y	Y	Y	–
Omar et al. [19]	U	N	N	N	Y	N	Per protocol analysis
Singh et al. [26]	U	N	N	N	Y	N	Per protocol analysis
Krogh et al. [14]	Y	Y	Y	Y	Y	Y	–
Creaney et al. [5]	U	Y	Y	Y	Y	Y	–
Raeissadat et al. [23]	Y	N	Y	N	Y	N	Per protocol analysis
Raeissadat et al. [24]	Y	N	N	N	Y	N	Per protocol analysis

### Direct comparisons

Data for direct comparisons of all treatments and outcomes measured at the end of each study are given in Table 1. Pooling according to outcomes was performed if there were at least two studies for each comparison, as clearly described below. There was no evidence of publication bias by Egger’s test for both pooled effects of all outcomes from direct comparison.

#### Visual analog score

In seven studies [6, 12, 19, 21, 23, 24, 33], the UMD of −1.7 (95 % CI −2.6, −0.8) and −2.5 (95 % CI −3.5, −1.5) showed that there was significantly lower VAS for PRP and AB, respectively, than for steroids (Table 3). The UMD was homogeneous (*I*^2^ = 0) with a value of −1.1 (95 % CI −1.3, −0.8), showing that VAS was significant lower for PRP than AB.

**Table 3:** Summarized results of direct comparisons according to type of interventions

Clinical outcomes	No. of studies	*I* ^2^	No. of subjects	UMD (95 % CI)
VAS				
PRP vs. AB	3	0	72 vs. 72	−1.1 (−1.3, −0.8)*
PRP vs. steroid	2	77.4	66 vs. 64	−1.7 (−2.6, −0.8)*
AB vs. steroid	2	0	60 vs. 60	−2.5 (−3.5, −1.5)*
DASH score				
PRP vs. steroid	2	91.6	96 vs. 94	−16.3 (−22.3, −10.4)*
AB vs. steroid	1	–	30 vs. 30	−25.5 (−33.8, −17.2)*
PRTEE score				
PRP vs. AB	1	–	80 vs 70	−11.0 (−18.3, −3.7)*
PRP vs. steroid	1	–	20 vs. 20	−7.3 (−13.8, −0.9)*
AB vs. steroid	1	–	30 vs. 30	−5.3 (−9.1, −1.6)*
PPT				
PRP vs. AB	2	68	58 vs. 58	2.5 (−1.5, 6.5)
AB vs. steroid	1	–	30 vs. 30	9.9 (5.6, 14.2)*

Adverse effects	No. of studies	*I* ^2^	No. of subjects	RR (95 % CI)
PRP vs. AB	1	–	14 vs.14	0.44 (0.17, 1.11)
PRP vs. steroid	2	0	71 vs. 69	1.00 (0.31, 3.24)
AB vs. steroid	2	0	60 vs. 60	1.78 (1.00, 3.17)*

Non-response rate	No. of studies	*I* ^2^	No. of subjects	RR (95 % CI)
PRP vs. steroid	1	–	51 vs. 49	1.23 (1.01, 1.49)*

*PRP* platelet-rich plasma, *AB* autologous blood, *Steroid* corticosteroid, *VAS* visual analog score, *DASH* Disabilities of the Arm Shoulder and Hand, *PRTEE* Patient-Rated Tennis Elbow Evaluation, *PPT* pressure pain threshold, *I* ^2^ degree of heterogeneity, *UMD* unstandardized mean differences, *CI* confidence interval, *RR* relative risk

* Statistically significant difference (*P* < 0.05)

#### Disabilities of the Arm Shoulder and Hand score

In three studies [12, 19, 21], the UMD of −16.3 (95 % CI −22.3, −10.4) and −25.5 (95 % CI −33.8, −17.2) showed that there was a significantly lower DASH score for PRP and AB, respectively, than for steroids (Table 3).

#### Patient-Related Tennis Elbow Evaluation score

In three studies [5, 14, 26], the UMD of −7.3 (95 % CI −13.8, −0.9) and −5.3 (95 % CI −9.1, −1.6) showed that there was a significantly lower PRTEE score for PRP and AB, respectively, than for steroids (Table 3). The UMD of −11.0 (95 % CI −18.3, −3.7) showing that the PRTEE score was significant lower for PRP than AB.

#### Pressure pain threshold

In three studies [12, 23, 24], the UMD of 9.9 (95 % CI 5.6, 14.2) showed that there was a significantly higher PPT score for AB than steroids (Table 3). The UMD of 2.5 (95 % CI −1.5, 6.5) showing that PPT was higher for PRP than AB, but this was not significant.

#### Adverse effects (local pain and skin reaction) and non-response rates

In five studies [6, 12, 14, 21, 33], the pooled RR was 1.78 (95 % CI 1.00, 3.17), which showed a significantly higher risk of complications after AB injection when compared with steroids, and no heterogeneity (*I*^2^ = 0) was present (Table 3). Compared with PRP, the pooled RR for AB and steroids had no statistically significant difference. Only one study [21] reported non-response rates. The pooled RR was 1.23 (95 % CI 1.01, 1.49), which showed a significantly higher risk of non-response after PRP injection when compared with steroid injection.

### Network meta-analysis

#### Visual analog score

**Fig. 2:**
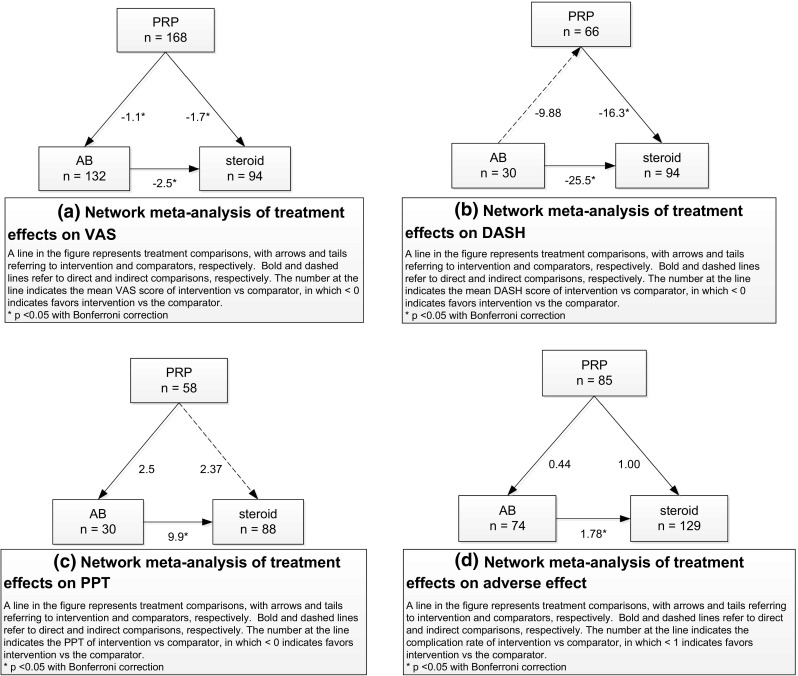
**a** Network meta-analysis of effects of treatment on VAS, **b** network meta-analysis of effects of treatment on DASH score, **c** network meta-analysis of effects of treatment on PPT, **d** network meta-analysis of effects of treatment on adverse effects

Seven studies [6, 12, 19, 21, 23, 24, 33] were included in the network meta-analysis. After being adjusted by time, the regression analysis suggested that for assessment within 2 months, the mean differences in VAS for PRP and AB showed that the VAS was lower than for steroid injection, but these were not significantly different (as seen in Table 4; Fig. 2a). For assessment at the last follow-up, the mean difference in VAS for PRP and AB injection was lower, with statistical significance, than for steroid injection.

**Table 4:** Comparisons of treatment effects: a network meta-analysis

Treatment	Within 2 months				At last follow-up			
	*N*	Mean	95 % CI	*P* value	*N*	Mean	95 % CI	*P* value
VAS								
PRP	153	3.64	2.84, 4.45	<0.001*	168	2.27	1.51, 3.02	<0.001*
AB	132	2.99	2.19, 3.80	<0.001*	132	2.90	2.09, 3.70	<0.001*
Steroid	79	4.18	3.04, 5.33,	<0.001*	94	4.29	3.31, 5.27	<0.001*

Treatment	Within 2 months				At last follow-up			
	*N*	Mean difference	95 % CI	*P* value	*N*	Mean difference	95 % CI	*P* value
VAS								
PRP vs. steroid	–	−0.54	−1.76, 0.68	0.386	–	−2.02	−3.04, −1.01	<0.001*
AB vs. steroid	–	−1.19	−2.41, 0.03	0.056	–	−1.39	−2.48, −0.30	0.012*
PRP vs. AB	–	0.65	−0.21, 1.51	0.138	–	−0.63	−1.47, 0.20	0.138

Treatment	Within 2 months				At last follow-up			
	*N*	Mean	95 % CI	*P* value	*N*	Mean	95 % CI	*P* value
DASH								
PRP	51	46.15	35.37, 56.93	<0.001*	66	17.38	8.42, 26.33	<0.001*
AB	30	7.49	−7.61, 22.59	<0.001*	30	7.49	−7.61, 22.59	<0.001*
Steroid	79	31.76	22.81, 40.71	<0.001*	94	35.95	28.10, 43.80	<0.001*

Treatment	Within 2 months				At last follow-up			
	*N*	Mean difference	95 % CI	*P* value	*N*	Mean difference	95 % CI	*P* value
DASH								
PRP vs. steroid	–	14.39	1.77, 27.00	0.025*	–	−18.58	−29.08, −8.08	0.001*
AB vs. steroid	–	−24.27	−40.68, −7.86	0.004*	–	−24.27	−40.68, −7.86	0.004*
PRP vs. AB	–	38.66	20.48, 56.83	<0.001*	–	9.88	−7.32, 27.08	0.260

Treatment	Within 2 months				At last follow-up			
	*N*	Mean	95 % CI	*P* value	*N*	Mean	95 % CI	*P* value
PRTEE								
PRP	100	36.37	24.09, 48.66	<0.001*	100	29.31	17.03, 41.60	<0.001*
AB	100	34.12	21.84, 46.41	<0.001*	100	33.87	21.59, 46.16	<0.001*
Steroid	50	30.82	18.53, 43.11	<0.001*	50	32.53	20.24, 44.82	<0.001*

Treatment	Within 2 months				At last follow-up			
	*N*	Mean difference	95 % CI	*P* value	*N*	Mean difference	95 % CI	*P* value
PRTEE								
PRP vs. steroid	–	5.55	−6.65, 17.76	0.373	–	−3.22	−15.42, 8.99	0.605
AB vs. steroid	–	3.30	−8.90, 15.51	0.596	–	1.34	−10.86, 13.55	0.829
PRP vs. AB	–	2.25	−9.96, 14.46	0.718	–	−4.56	−16.77, 7.65	0.464

Treatment	Within 2 months				At last follow-up			
	*N*	Mean	95 % CI	*P* value	*N*	Mean	95 % CI	*P* value
PPT								
PRP	58	18.58	12.66, 24.51	<0.001*	58	20.03	14.11, 25.96	<0.001*
AB	30	21.23	15.16, 27.31	<0.001*	30	27.53	21.46, 33.61	<0.001*
Steroid	88	17.57	11.70, 23.44	<0.001*	88	17.67	11.80, 23.54	<0.001*

Treatment	Within 2 months				At last follow-up			
	*N*	Mean difference	95 % CI	*P* value	*N*	Mean difference	95 % CI	*P* value
PPT								
PRP vs. steroid	–	1.02	−0.48, 2.52	0.184	–	2.37	0.87, 3.87	0.02*
AB vs. steroid	–	3.67	1.64, 5.69	<0.001*	–	9.87	7.84, 11.89	<0.001*
PRP vs. AB	–	−2.65	−5.00, −0.30	<0.001	–	−7.50	−9.85, −5.15	<0.001*

Treatment	Within 2 months				At last follow-up			
	*N*	IR	95 % CI	*P* value	*N*	IR	95 % CI	*P* value
Adverse effects								
PRP	–	–	–	–	85	0.10	0.03, 0.34	<0.001*
AB	–	–	–	–	74	0.20	0.06, 0.65	0.008*
Steroid	–	–	–	–	129	0.11	0.03, 0.35	<0.001*

Treatment	Within 2 months				At last follow-up			
	*N*	RR	95 % CI	*P* value	*N*	RR	95 % CI	*P* value
Adverse effects								
PRP vs. steroid	–	–	–	–	–	0.90	0.36, 2.24	0.821
AB vs. steroid	–	–	–	–	–	1.88	0.95, 3.72	0.068
PRP vs. AB	–	–	–	–	–	0.004	0.0002, 0.09	0.001*

*PRP* platelet-rich plasma, *AB* autologous blood, *Steroid* corticosteroid, *VAS* visual analog score, *DASH* Disabilities of the Arm Shoulder and Hand, *PRTEE* Patient-Rated Tennis Elbow Evaluation, *PPT* pressure pain threshold, *CI* confidence interval, *IR* incident rate, *RR* relative risk

* Statistically significant difference (*P* < 0.05)

#### Disabilities of the Arm Shoulder and Hand score

Three studies [12, 19, 21] were included in the network meta-analysis. After being adjusted for time frame, the regression analysis suggested that for assessment within 2 months, the mean difference in DASH score for AB injection was statistically significantly lower than for PRP and steroid injection, with a value of −38.66 (95 % CI −56.83, 20.48) and −24.27 (95 % CI −40.68, 7.86), respectively (Table 4; Fig. 2b). However, assessment at the last follow-up of AB injection was statistically significantly lower than steroid injection but not significantly different when compared with PRP.

#### Patient-Related Tennis Elbow Evaluation score

Data from three studies [5, 14, 26] were included in the network meta-analysis of PRTEE score (Table 4). The lowest mean PRTEE scores were for steroid injection and PRP injection with a value of 30.82 (95 % CI 18.53, 43.11) and 29.31 (95 % CI 17.03, 41.60) when assessed within 2 months and at most recent follow-up, respectively. There was no significant difference between the two active treatments (Table 4).

#### Pressure pain threshold

Data from three studies [12, 23, 24] were included in the network meta-analysis of PPT (Table 4). The highest mean PPT was for AB injection with a value of 21.23 (95 % CI 15.16, 27.31) and 27.53 (95 % CI 21.46, 33.61) when assessed within 2 months and at last follow-up, respectively. The regression analysis suggested that the mean difference in PPT for AB injection was statistically significantly higher than for PRP and steroid injection with a value of 2.65 (95 % CI 0.30, 5.00) and 3.67 (95 % CI 1.64, 5.69) when assessed within 2 months and at last follow-up assessment, respectively; the mean difference between PRP and AB was statistically significant and increased to 7.50 (95 % CI 5.15, 9.85) and 9.87 (95 % CI 7.84, 11.89) (Table 4; Fig. 2c).

#### Adverse effects (local pain and skin reaction) and non-response rates

Data from five studies [6, 12, 14, 21, 33] were included in the network meta-analysis. Compared to AB injection, PRP and steroid injection had lower risks of having complications, with borderline statistical significance of 99.6 % (RR = 0.004; 95 % CI 0.0002, 0.09) and 53 % (RR = 0.53; 95 % CI 0.27, 1.05), respectively. PRP injection had an approximately 10 % (RR = 0.90; 95 % CI 0.36, 1.27), statistically not significant, lower risk than steroid injection (Table 4; Fig. 2d).

## Discussion

The result of the present study was that PRP injection significantly improves pain and PRTEE score when compared with AB injection and steroid injection. Compared to AB injection, steroid injection had significantly improved disability score (DASH) and significantly improved pressure pain threshold (PPT). The chances of adverse effects from PRP injection and steroid injection were not significantly different but AB injection had a significantly higher chance of adverse effects when compared with steroid injection. Multiple active treatment comparisons with time adjustment indicated that within 2 months only AB injection showed an improvement of borderline significance (0.0056) in pain VAS, but PRP and AB injection showed a significant improvement in pain VAS when compared with steroid injections. AB injection had significantly improved DASH scores and PPT when compared with PRP and steroid injections, but AB injection had a statistically significantly higher risk of adverse effects when compared with PRP and steroid injections at the last follow-up assessment. For PRTEE score, there was no significant difference between the two active treatments.

The results of this study were consistent with previous meta-analyses by Ahmad et al. [1] which showed that PRP was more efficacious than AB injection in terms of pain VAS, and that PRP was more efficacious than steroid injections in terms of pain VAS. There is additional evidence with good methodological quality (RCT) that PRP injection and AB injection displays an improvement in disability scores (DASH, PRTEE) and pressure pain threshold (PPT) when compared with steroid injection. However, the highest risk of having adverse effects was with AB injection when compared with PRP and steroid injections.

The direct meta-analysis suggests potential benefits of AB injection in reducing pain, improving disabilities scores and pressure pain threshold, but increasing the risk of adverse effects when compared with steroids, whereas PRP injection can reduce pain, improve disabilities scores and pressure pain threshold, but has increased rates of non-response after injection when compared with steroid injections. However, for other outcomes there was no significant difference. There are limitations of direct meta-analysis from the small number of studies that evaluated each particular pair of treatments, but a network meta-analysis circumvents this problem by creating indirect comparisons between active treatments and difference in time of assessment that can identify the most effective therapy and the time period that is the most beneficial. In this case, AB injection was the best therapy at the assessment times of within 2 months and over 2 months for improvement of DASH score and PPT, as during the second time period it had a cumulative effect. AB injection may be the worst therapy in terms of risks of adverse effects when compared with PRP and steroid injections. None of the RCTs compared combined treatments with AB injection or PRP injection and steroid injection.

This study has several strengths. A network meta-analysis was applied to increase the power of the tests and reduce Type I errors. A regression model was used, taking into account study effects in order to assess treatment effects. The network meta-analysis ‘borrows’ treatment information from other studies and increases the total sample size. As a result, treatment effects that could not be detected in direct meta-analysis could be identified. All possible treatment comparisons are mapped and displayed in Table 5. Although the pooled estimates were heterogeneous, the regression model with cluster effect takes variations at the study level into account. The limitations recognized in this review are that some pooled results were heterogeneous but the source of heterogeneity was not explored due to limitations in the reported data.

**Table 5:** Summary of all treatment effects for lateral epicondylitis patients

Treatments	Pain VAS	DASH score	PRTEE score	PPT	Adverse effects	Non-response rate
PRP vs. AB	(D* & N)	(N*)	(D* & N)	(D & N*)	(D & N*)	–
PRP vs. steroid	(D* & N*)	(D* & N*)	(D* & N)	(N*)	(D & N)	(D*)
AB vs. steroid	(D* & N*)	(D* & N*)	(D* & N)	(D* & N*)	(D* & N)	–

*D* direct, *N* network

* Statistically significant difference (*P* < 0.05)

Based on the evidence presented, it can be concluded that when comparing three active treatments, PRP injection was the best treatment for reducing pain VAS after 2 months whereas AB injection was the best treatment for improving disabilities scores (DASH, PRTEE) and increasing pressure threshold (PPT) both within and after 2 months. However, AB injection had the highest risk of adverse effects (injection site pain and skin reaction). Further research should be done regarding cost-effective analysis comparing PRP injection and AB injection or the combination of AB injection and multi-modality physical therapy, possibly improving outcomes for pain, disabilities scores, and pressure pain threshold as well as lowering the risk of adverse effects.

In conclusion, this network meta-analysis has provided additional information that PRP injection or AB injection can be selected for management of chronic lateral epicondylitis. PRP can improve pain and lower the risk of adverse effects whereas AB injection can improve pain, disabilities scores and pressure pain threshold but has a higher risk of adverse effects.
